# Infection in Living Donor Liver Transplantation Leads to Increased Risk of Adverse Renal Outcomes

**DOI:** 10.3390/nu14173660

**Published:** 2022-09-04

**Authors:** Kao-Ming Hsu, Pei-Ru Lin, Ping-Fang Chiu, Yao-Peng Hsieh

**Affiliations:** 1 Division of Nephrology, Department of Internal Medicine, Changhua Christian Hospital, Changhua 50006, Taiwan; 2 Big Data Center, Changhua Christian Hospital, Changhua 50006, Taiwan; 3 Department of Hospitality Management, MingDao University, Changhua 52345, Taiwan; 4 Department of Post Baccalaureate Medicine, College of Medicine, National Chung Hsing University, Taichung 40227, Taiwan; 5 School of Medicine, Kaohsiung Medical University, Kaohsiung 80708, Taiwan; 6 School of Medicine, Chung Shan Medical University, Taichung 40201, Taiwan

**Keywords:** chronic kidney disease, infection, liver transplantation, pneumonia

## Abstract

(1) Background: Little is known about the subsequent renal function change following incident infectious diseases in living-donor liver transplant (LT) recipients. (2) Methods: We studied patients who underwent living-donor LT from January 2003 to January 2019 to evaluate the association of incident hospitalization with major infections or pneumonia with adverse renal outcomes, including a sustained 40% reduction in estimated glomerular filtration rate (eGFR) and renal composite outcome (a 40% decline in eGFR, end-stage renal disease, or death.). Multivariable-adjusted time-dependent Cox models with infection as a time-varying exposure were used to estimate hazard ratio (HR) with 95% confidence interval (CI) for study outcomes. (3) Results: We identified 435 patients (mean age 54.6 ± 8.4 years and 76.3% men), of whom 102 had hospitalization with major infections during follow-up; the most common cause of infection was pneumonia (38.2%). In multiple Cox models, hospitalization with a major infection was associated with an increased risk of eGFR decline > 40% (HR, 3.32; 95% CI 2.13–5.16) and renal composite outcome (HR, 3.41; 95% CI 2.40–5.24). Likewise, pneumonia was also associated with an increased risk of eGFR decline > 40% (HR, 2.47; 95% CI 1.10–5.56) and renal composite outcome (HR, 4.37; 95% CI 2.39–8.02). (4) Conclusions: Our results illustrated the impact of a single infection episode on the future risk of adverse renal events in LT recipients. Whether preventive and prophylactic care bundles against infection and judicious modification of the immunosuppressive regimen benefit renal outcomes may deserve further study.

## 1. Introduction

Significant improvements in long-term survival in liver transplant (LT) recipients are attributed to advances in surgical techniques, perioperative care, post-LT immunosuppression, and ideal management of postoperative complications. However, chronic kidney disease (CKD) remains a common complication after LT and affects 20%−50% of LT recipients, depending on the follow-up period and the definition of CKD stage [1,2,3]. Although the majority of them are stage 3 CKD, progressive renal fibrosis and parenchymal damage do eventually lead to renal replacement therapy, contributing to morbidity and mortality after LT. Contemporary literature has assessed risk factors associated with CKD after LT in an attempt to reduce its incidence [4].

Despite advanced development of immunosuppressive agents, infection still significantly contributes to morbidity and mortality and is the leading cause of readmission after LT [5]. Infectious processes also play a pivotal role in the development of CKD, as evidenced by animal studies demonstrating that infection could cause renal fibrosis, ischemic injury and affect the cellular composition of unstable atherosclerotic plaques, resulting in kidney dysfunction [6,7,8,9]. Besides, infection initiates many of the autoimmune or other reactions in genetically susceptible individuals [10]. However, only a few previous studies have assessed the association of infection with subsequent renal dysfunction [11,12,13,14,15].

LT recipients are susceptible to CKD and infection due to comorbidities and immunosuppressive agents. Pneumonia, the most common type of post-LT pulmonary complication, leads to more than 50% mortality [16]. To date, no studies have assessed the association of infection with renal outcomes in LT. We conducted the present study to test the hypothesis that hospitalization with a major infection would increase the subsequent risk of renal function decline in LT recipients.

## 2. Materials and Methods

### 2.1. Participants and Measurements

Participants included patients with liver failure or hepatocellular carcinoma who met the Milan criteria for liver transplantation and underwent living-donor liver transplantation at Changhua Christian Hospital between January 2003 and January 2019. Informed consent was exempted for a retrospective observational study in Taiwan and the institutional review board approved the study which was conducted in accordance with the declaration of Helsinki.

Covariates assessed at baseline included age, gender, comorbidity, Charlson comorbidity index (CCI) and laboratory data. The index date was defined as the date when stable renal function resumed after surgery. eGFR was calculated using the abbreviated 4-variable Modification of Diet in Renal Disease (MDRD) formula: estimated GFR mL/min per 1.73 m^2^ = 186 × serum creatinine^−1.154^ × age^−0.203^ × 0.742 (if female patient) × 1.212 (if African American) [17].

### 2.2. Exposures of Interest

The primary exposure of interest was incident hospitalization with major infection after the index date, including pneumonia, urinary tract infection, infectious enterocolitis, primary bacteremia, and soft-tissue infection. The most common type of infection was pneumonia in our study cohort. Incident hospitalization with pneumonia was designated as a secondary exposure to assess its association with our study outcomes.

### 2.3. Outcomes of Interest

Surrogate endpoints were used because the enrolled patients had chronic slow renal progression patterns. The primary outcome of interest was a sustained 40% reduction in eGFR. The secondary outcome of interest was the renal composite outcome of a 40% decline in eGFR, ESRD, or death.

### 2.4. Statistical Analysis

Baseline data were presented as mean ± standard deviation (SD) or number with percentage for continuous and categorical variables, respectively. Student’s *t*-test and Chi-square test were used to compare the distribution of continuous and categorical variables between patients who did and did not have hospitalization with major infection, as appropriate.

Incident major infection after the index date was classified as a time-updated exposure. Thus, if a patient developed infection during follow-up, they contributed person-time to the non-infection exposure group until they had incident hospitalization with infection when they began contributing person-time to the infection exposure group for the remaining follow-up period.

The Simon and Makuch’s modified Kaplan–Meier curves were plotted and calculated for comparison of cumulative renal outcomes between patients with and without infection and the Mantel–Byar test was used to compare the curve differences between the two groups. Given the immortal bias and competing risk of death in infection exposure group, a time-dependent Cox model using Fine and Gray’s method was used to assess the association between infection and study outcomes. Patients were censored at occurrence of outcome events, death or the end of study on 31 May 2020. Hazard ratios (HRs) with 95% confidence intervals (CI) were presented in all models. Variables with significant contributions (*p* < 0.05) in univariate Cox models were incorporated into the multiple adjusted Cox models to determine the independent association with study outcomes. All statistical analyses were carried out using the statistical software package SPSS (IBM SPSS Statistics, version 20, IBM Corporation, Armonk, NY, USA). Finally, a two-sided *p* < 0.05 was considered statistically significant.

## 3. Results

### 3.1. Baseline Characteristics

The study cohort included 435 patients who met the eligibility criteria and were divided into two groups based on the occurrence of incident hospitalization with major infections following the index date. Of these patients, 102 developed major infections, whereas the remaining 333 did not. Table 1 summarizes the demographics of the two groups, comparing patients with infections with those without infection. The most common cause of infection was pneumonia (*n* = 39; 38.2%). No significant differences between the two groups were observed in various comorbid conditions, including diabetes, hypertension, cirrhosis, coronary artery disease, congestive heart failure, and hepatitis B and C. Patients with infection were more likely to be of female gender and higher CCI than those without infection. There was no significant difference in baseline laboratory parameters between the two groups, except for higher CRP in the infection group. The mean eGFR at the index date in all patients was 77.2 ± 29.5 mL/min/1.73 m^2^, comprising 80.5 ± 36.7 and 76.1 ± 26.9 mL/min/1.73 m^2^ for the infection group and non-infection group (*p* value = 0.266), respectively.

### 3.2. Association of Infection with Renal Outcomes during the Follow-Up Period

**Figure 1:**
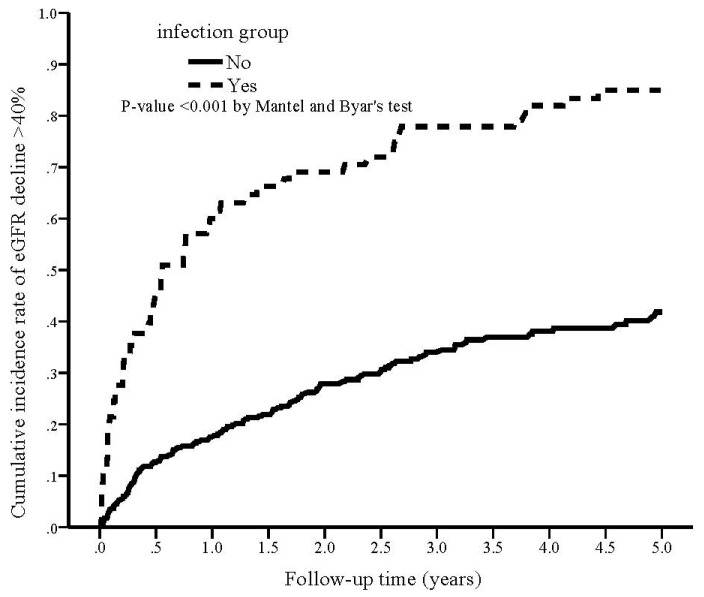
Kaplan–Meier curve of the cumulative incidence rate of eGFR decline > 40% for patients with and without infection (Mantel and Byar’s test, *p* < 0.001).

During the study period, 74 (72.5%) patients in the infection group reached eGFR decline > 40% compared with 97 (29.1%) in the non-infection group (*p* < 0.001; Table 2). The cumulative rate of eGFR decline > 40% was plotted and compared using the Simon and Makuch methods (Figure 1). Infection group was significantly associated with a higher cumulative rate of eGFR decline > 40% compared with non-infection group (*p* < 0.001 by Mantel and Byar’s test). The results of unadjusted and adjusted competing risk regression analyses of the incidence of eGFR decline > 40% are shown in Table 3. The crude HR of developing eGFR decline > 40% was 3.51 (95% CI 2.38–5.17, *p* < 0.001) for the infection group. After fully adjusting for confounding factors (model 4), the adjusted HR of developing eGFR decline > 40% in the infection group was 3.32 (95% CI 2.13–5.16, *p* < 0.001) as compared with non-infection.

**Figure 2:**
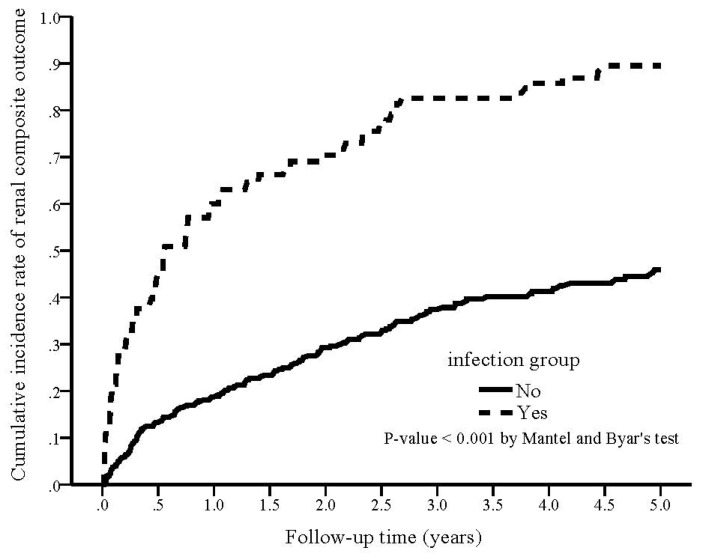
Kaplan–Meier curve of the cumulative incidence rate of renal composite outcome for patients with and without infection (Mantel and Byar’s test, *p* < 0.001).

During the study period, 80 (78.4%) patients in the infection group reached the renal composite outcome compared with 111 (33.3%) in the non-infection group (*p* < 0.001; Table 2). The cumulative rate of renal composite outcome was plotted and compared using the Simon and Makuch methods (Figure 2), demonstrating a significantly higher cumulative rate of renal composite outcome in the infection group (*p* < 0.001 by Mantel and Byar’s test). The crude HR of developing a renal composite outcome was 3.69 (95% CI 2.66–5.13, *p* < 0.001) for the infection group. After adjusting for confounding factors, the adjusted HR of developing a renal composite outcome in the infection group was 3.41 (95% CI 2.40–4.86, *p* < 0.001) as compared with non-infection.

### 3.3. Association of Pneumonia with Renal Outcomes during the Follow-Up Period

**Figure 3:**
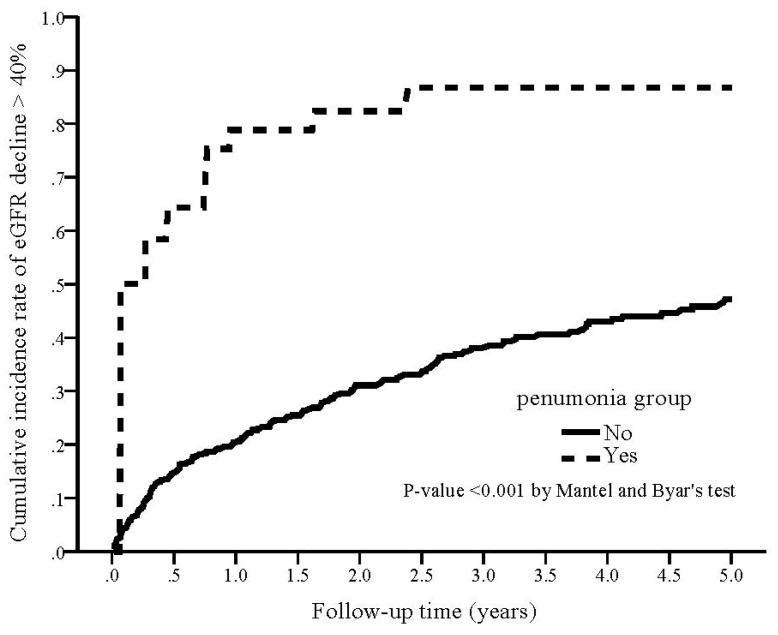
Kaplan–Meier curve of the cumulative incidence rate of eGFR decline > 40% for patients with and without pneumonia (Mantel and Byar’s test, *p* < 0.001).

During the study period, 29 (74.4%) patients in the pneumonia group reached eGFR decline > 40% compared with 142 (35.9%) in the non-pneumonia group (*p* < 0.001; Table 2). The cumulative rate of eGFR decline > 40% was plotted and compared using the Simon and Makuch methods (Figure 3). The pneumonia group was significantly associated with a higher cumulative rate of eGFR decline > 40% compared with the non-pneumonia group (*p* < 0.001 by Mantel and Byar’s test). The results of unadjusted and adjusted competing risk regression analyses of the incidence of eGFR decline > 40% were showed in Table 2. The crude HR of developing eGFR decline > 40% was 2.73 (95% CI 1.28–5.82, *p* < 0.001) for the pneumonia group. After adjusting for confounding factors, the adjusted HR of developing eGFR decline > 40% in the pneumonia group was 2.47 (95% CI 1.10–5.56, *p* = 0.029) as compared with non- pneumonia.

**Figure 4:**
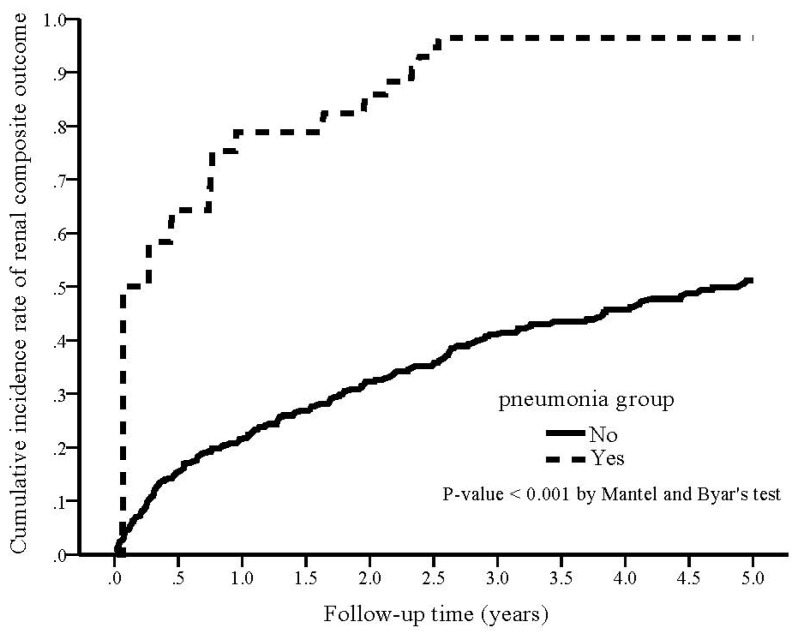
Kaplan–Meier curve of the cumulative incidence rate of renal composite outcome for patients with and without pneumonia (Mantel and Byar’s test, *p* < 0.001).

During the study period, 34 (87.2%) patients in the pneumonia group reached the renal composite outcome compared with 142 (35.9%) in the non-pneumonia group (*p* < 0.001). The cumulative rate of renal composite outcome was plotted and compared using the Simon and Makuch methods (Figure 4), demonstrating a significantly higher cumulative rate of renal composite outcome in the pneumonia group (*p* < 0.001 by Mantel and Byar’s test). The crude HR of developing a renal composite outcome was 5.07 (95% CI 2.94–8.77, *p* < 0.001) for the pneumonia group. After adjusting confounding factors, the adjusted HR of developing a renal composite outcome in the pneumonia group was 4.37 (95% CI 2.39–8.02, *p* < 0.001) as compared with non-pneumonia.

## 4. Discussion

Infection is common in patients undergoing LT, but few studies that have addressed the clinical consequences of infection have primarily focused on patient and graft survival. Likewise, CKD is also a common complication in LT recipients and significantly affects overall survival. Animal and epidemiologic studies have provided evidence that infection could lead to renal function decline in the short and long term through a variety of pathophysiologic mechanisms. Pneumonia, one of the most common infections after LT, was associated with prolonged use of mechanical ventilation and the need for renal replacement therapy [18]. To the best of our knowledge, our study is the first one to investigate the impact of infection on renal events in living donor LT. In the present cohort study of 435 living donor LT recipients, hospitalization with major infection or pneumonia was independently and robustly associated with a higher risk of adverse renal outcomes, including a sustained 40% reduction in eGFR and renal composite outcome.

Few previous studies reported a significantly higher risk of infection with subsequent renal outcomes. An infection episode was independently associated with an increased risk of ESRD or mortality in patients with advanced CKD [12]. A recent prospective cohort study of 10,290 participants from 4 US communities associated any hospitalization for major infection with a higher risk of ESRD [14]. Our findings were consistent with theirs and showed a significantly higher risk of renal function decline following any hospitalization with major infection in the setting of LT after controlling for many confounders of CKD development. Regarding the impact of pneumonia, the most common type of infection in our cohort, a limited number of previous studies have investigated its association with CKD outcomes. A Taiwanese study using the National Health Insurance database showed that pneumococcal pneumonia was associated with ESRD in adult patients [13]. Associations between hospitalization with pneumonia and raised risk of subsequent CKD in adulthood were also confirmed by Sundin et al. [11]. Similar findings were also documented by Su et al. and Ishgami et al. who examined the outcomes of CKD progression and incident ESRD in the CKD cohort and general population, respectively [14,15]. Our results also indicated a higher risk of adverse renal outcomes following pneumonia episodes in LT recipients.

Although the causality cannot be established in our cohort study, the associations of infection and adverse renal outcomes can be explained by several plausible biological pathways. The mechanisms through which infection results in adverse renal outcomes may involve the exaggerated inflammatory processes and comorbidities burden. In our study, patients experiencing an infectious episode had more comorbidities (a higher CCI) and higher CRP, both of which can result in a higher risk of renal dysfunction. An infective event may stimulate or exacerbate these inflammatory pathways, leading to worse renal outcomes. In stepwise model adjustments, the risk for study outcomes attenuated slightly but remained statistically significant. This suggested that the burden of comorbidities and high CRP only partially contributed to and did not completely explain the higher renal risk. Infection-induced episodes of hypotension and myocardial dysfunction can lead to tissue hypoxia [19]. In addition, excessive fluid resuscitation to maintain adequate perfusion pressure can also cause renal congestion and impede renal perfusion [20]. Furthermore, various diagnostic or therapeutic interventions can also contribute to renal dysfunction. For example, the use of antibiotics (e.g., aminoglycosides) and radioactive contrast agents can predispose to renal tubular and interstitial damage [17,21]. Finally, AKI during infection is another postulated mechanism because it is a well-established independent risk factor for CKD progression [22].

Previous studies have provided plausible biological mechanisms specific to pneumonia affecting renal adverse events. Intriguingly, inflammatory cytokines after pneumonia remained elevated throughout the first week and beyond resolution of clinical signs of infection, and persistent inflammation may contribute to worsening renal function [23,24,25]. Systemic hypoxia may result in peritubular hypoxia, leading to tubulointerstitial fibrosis in the presence of various cytokines [26]. Finally, a prothrombotic milieu, such as higher levels of soluble P-selectin and thromboxane B2 through marked platelet activation and hyper-reactivity in pneumonia, is predictive of renal function decline [27,28,29].

Overall, this study has several clinical implications and demonstrates the need to understand renal consequences following infection in the LT population. Immunosuppressive agents should be used to balance transplant rejection and susceptibility to infection because infectious complications are undisputed sequelae of these medications. Given that individual responses to specific immunosuppressive regimens vary, careful and personalized modification of immunosuppressive regimens may optimize anti-rejection efficacy and reduce infection risk. Despite the lack of causality, our findings here evidence the need for vaccination campaigns and guidance on infection prevention precautions. For example, pneumococcal and influenza vaccines have been recommended as prevention approaches in the guidelines for adults aged 19 or older [30]. In addition, the effective preventive and prophylactic care bundles should include strict environmental infection control measures, universal precautions, and hand hygiene practices [31]. 

Previous literature examining the association of infection and renal outcomes primarily has investigated selected populations of CKD, military conscripted men or US community residents, or has used the national registry data [11,12,13,14,15]. We extended this to living donor LT patients and confirmed that any hospitalization with major infection irrespective of severity was an independent risk factor for increased adverse renal events in the LT recipients. Strengths of the present study include its moderate sample size, vigorous definition of renal function decline by a sustained eGFR decline > 40%, validation of medical diagnosis from medical records and use of time-dependent death-competing Cox regression models, which account for immortal bias for the infection group because patients must survive long enough to develop an infection.

Like other observational studies, our results should be interpreted with limitations. First, the presence of unknown or residual confounders is inherent in retrospective observational studies, and associations may attenuate or disappear when unidentified key causal factors mediating infection and renal events are included. For example, we did not collect information on infection severity, nephrotoxic medications, ICU admission or renal replacement therapy during the index hospitalization. However, we extensively collected and accounted for known CKD risk factors and multivariate adjustments confirmed that the robust associations were independent. Nonetheless, future researches are required to explore the mediators linking infection to the subsequent renal events as knowledge of true mediators has clinical implications for CKD prevention. Second, we only included major infections requiring hospitalization. Thus, whether infections in the outpatient care were associated with renal function decline warrants future studies. However, since the reliability of outpatient infection diagnosis is unclear, more caution is required when conducting these studies in outpatient settings. Our focus on hospitalization with acute infection remains valuable from a public health perspective because of its significant impact on morbidity and mortality. Third, a single-center study design of patients undergoing living donor LT limits its generalization to other racial populations or deceased donor LT. Fourth, we aimed to study whether acute infection (single episode) was associated with adverse renal events in the present study. How the frequency of infection episodes affects the long-term renal outcomes warrants further investigation. Finally, since our study cohort was restricted to living donor LT, our results may not be applicable to deceased donor LT. We believe these limitations will not invalidate our novel findings.

## 5. Conclusions

In conclusion, the present study indicated that hospitalization with major infection was common amongst LT recipients and the most common cause of infection was pneumonia. Hospitalization with any major infection or pneumonia irrespective of its severity was independently associated with subsequent adverse renal outcomes, including a sustained 40% reduction in eGFR and renal composite outcome. Our findings are consistent with previous literature demonstrating the adverse sequelae of infection episodes on kidney function. LT recipients who experienced an episode of infection should be considered at high risk for CKD during follow-up. Thus, long-term careful monitoring of renal function is recommended in LT recipients with any major infection episode. Future investigations should focus on examining mechanisms by which the infection leads to renal function decline and whether infection prevention strategies for preventable infection help reduce the occurrence of CKD.

## Figures and Tables

**Table 1:** Patient demographics.

	With Infection	Without Infection	*p*-Value
Sample size	102	333	
Age (years)	55.1 ± 8.6	54.4 ± 8.3	0.456
Gender, male	68 (66.7%)	264 (79.3%)	0.009 *
Comorbidity			
CCI	4.6 ± 4.2	3.3 ± 3.3	0.001 *
DM	20 (19.6%)	47 (14.1%)	0.179
Hypertension	11 (10.8%)	33 (9.9%)	0.798
Hepatitis B	36 (35.3%)	119 (35.7%)	0.935
Hepatitis C	26 (25.5%)	74 (22.2%)	0.493
Cirrhosis	52 (51%)	161 (48.3%)	0.642
CHF	9 (8.8%)	21 (6.3%)	0.380
CAD	0 (0%)	5 (1.5%)	0.213
Lab data at baseline			
BUN (mg/dL)	19.6 ± 15.6	17.2 ± 12.3	0.118
Creatinine (mg/dL)	1.15 ± 0.62	1.12 ± 0.74	0.733
eGFR (mL/min/1.73 m^2^)	76.1 ± 38.6	74.9 ± 29.9	0.748
Albumin (g/dL)	2.9 ± 0.8	2.9 ± 0.7	0.975
AST (U/L)	177 ± 94.5	167.2 ± 95.9	0.366
ALT (U/L)	110 ± 53.3	112.1 ± 68.2	0.774
PT (second)	18.4 ± 4.5	17.9 ± 4	0.287
aPTT (second)	38.2 ± 8.4	38.2 ± 10.2	0.953
Platelet (10^3^/μL)	89.1 ± 39.4	82.9 ± 38.9	0.167
CRP (mg/dL)	2.7 ± 4.5	0.7 ± 1.9	<0.001 *
Anti-HBs (mIU/mL)	85 (83.3%)	266 (79.9%)	0.439
Average tacrolmius concentration (ng/mL)	6.32 ± 2.5	6.34 ± 2.1	0.931

CCI: Charlson comorbidity index; CHF: congestive heart failure; CAD: coronary artery disease; BUN: blood urea nitrogen, AST: aspartate aminotransferase, ALT: alanine aminotransferase, PT: prothrombin time, aPTT: activated partial thromboplastin time, CRP: C-reactive protein; DM: diabetes mellitus; eGFR: estimated glomerular filtration rate; Anti-Hb Ab: hepatitis B surface antibody. *: *p* < 0.05.

**Table 2:** Comparisons of study outcomes by the presence/absence of infection and pneumonia.

	eGFR Decline > 40%	Renal Composite Outcome
(A) infection		
Yes	74 (72.5%)	80 (78.4%)
No	97 (29.1%)	111 (33.3%)
*p* value	<0.001	<0.001
(B) pneumonia		
Yes	29 (74.4%)	34 (87.2%)
No	142 (35.9%)	157 (39.6%)
*p* value	<0.001	<0.001

eGFR: estimated glomerular filtration rate.

**Table 3:** Stepwise Cox regression models of renal outcomes for infection/pneumonia groups.

	eGFR Decline > 40%		Composite Renal Outcomes	
	Hazard Ratio (95% CI)	*p*-Value	Hazard Ratio (95% CI)	*p*-Value
(A) Infection vs. non-infection				
Model 1	3.51 (2.38–5.17)	<0.001	3.69 (2.66–5.13)	<0.001
Model 2	3.44 (2.31–5.11)	<0.001	3.68 (2.63–5.13)	<0.001
Model 3	3.20 (2.13–4.81)	<0.001	3.48 (2.48–4.88)	<0.001
Model 4	3.32 (2.13–5.16)	<0.001	3.41 (2.40–4.86)	<0.001
(B) pneumonia vs. non-pneumonia				
Model 1	2.73 (1.28–5.82)	0.009	5.07 (2.94–8.77)	<0.001
Model 2	2.67 (1.25–5.73)	0.012	4.86 (2.80–8.45)	<0.001
Model 3	2.24 (1.01–4.97)	0.047	4.17 (2.36–7.38)	<0.001
Model 4	2.47 (1.10–5.56)	0.029	4.37 (2.39–8.02)	<0.001

Model 1: unadjusted. Model 2: age, sex. Model 3: Model 1 plus comorbidities. Model 4: Model 2 plus lab data.

## Data Availability

The data sets generated or analyzed during the current study are available from the corresponding author upon reasonable request.
